# Apparent prevalence and risk factors of coxiellosis (Q fever) among dairy herds in India

**DOI:** 10.1371/journal.pone.0239260

**Published:** 2020-09-15

**Authors:** Pankaj Dhaka, Satya Veer Singh Malik, Jay Prakash Yadav, Manesh Kumar, Sukhadeo B. Barbuddhe, Deepak B. Rawool

**Affiliations:** 1 Division of Veterinary Public Health, ICAR-Indian Veterinary Research Institute, Izatnagar, Bareilly, India; 2 ICAR-National Research Centre on Meat, Chengicherla, Hyderabad, India; National Veterinary School of Toulouse, FRANCE

## Abstract

*Coxiella burnetii* is a highly infectious zoonotic pathogen infecting wide range of mammals, including humans. In the present study, a total of 711 blood samples from bovines [cattle (n = 543) and buffaloes (n = 168)] from eight farms at different geographical locations in India were screened for *C*. *burnetii* targeting the *IS1111* and the *com1* genes. The anti-*C*. *burnetii* antibodies in serum samples were detected using indirect-ELISA kits. Also, a total of 21 parameters pertaining to animal health and farm management were identified to assess their role as possible risk factors for coxiellosis among the targeted farms. The apparent prevalence (positive for PCR and/or ELISA) for coxiellosis was reported to be 24.5% in cattle and 8.9% in buffaloes. In cattle, the detection rate of *C*. *burnetii* employing the *IS1111* gene (8.5%) was found to be significantly higher (p<0.05) as compared to the *com1* (6.5%) gene. The seropositivity by ELISA was higher among cattle (17.7%) than in buffaloes (8.3%). Further, on univariable analysis of risk factors, species (cattle) (OR:3.31; 95%CI:1.88–5.82), inadequate floor spacing (OR:1.64; 95%CI:1.10–2.43), mastitis (OR:2.35, 95%CI:1.45–3.81) and reproductive disorders (OR:2.54; 95%CI:1.67–3.85) were significantly (p<0.05) having high odds for coxiellosis. The multivariable logistic regression analysis of the animal level risk factors revealed that species and age were found to be significantly associated with coxiellosis. However, since the number of screened farms is limited; further research is needed with a higher number of animals to confirm the farm level odds ratio of risk factors. Quarantine and biosecurity measures including farm hygiene operations were observed to be inadequate and also the lack of awareness about coxiellosis among the farm workers. In absence of vaccination program for coxiellosis in India, robust surveillance, farm biosecurity measures and the awareness for the disease among risk groups can play an important role in the disease prevention and subsequent transmission of the pathogen.

## Introduction

Coxiellosis (also known as Q fever), a zoonosis of public health concern, is caused by Gram-negative intracellular bacterium *Coxiella burnetii* [1]. The disease is considered as endemic in more than 51 countries [2] but remains a largely ‘neglected zoonosis’ [3]. In addition, the disease has been ranked as the most contagious and listed as one among the 13 ‘global priority zoonoses’ [4]. In developing countries, the disease causes significant impact on public health as well as socio-economic structure of the animal husbandry sector. The prevalence in these countries has been reported around 25% and the infected animals are the major sources of infection to farmers and other contact groups [4]. The Netherlands outbreak (2007–2010) of Q fever provided a clear demonstration of the serious threat posed to the public health in the absence of adequate diagnostic, therapeutic and epidemiological tools [5].

*Coxiella burnetii* is considered as ubiquitous zoonotic contaminant [6]. The reservoir hosts for the disease are extensive which include mammals, birds, and arthropods. However, ruminants are considered as the major reservoirs and the disease in ruminants is generally known as coxiellosis [1, 7]. The disease in ruminants is frequently subclinical, but late abortions, stillbirths and reproductive disorders can occasionally be noticed [8, 9].

In developing countries, due to sparse availability of diagnostic facilities, limited number of epidemiological studies on *C*. *burnetii* had been carried out [10, 11]. The standard routine laboratory culture methods are not appropriate to grow the pathogen and the isolation procedures require biosafety level 3 (BSL3) facilities with appropriate personal protective equipment(s) [1]. Therefore, specific indirect diagnostic tools including, molecular detection by PCR assays in clinical samples and serological assays (e.g., ELISA) for the detection of specific antibodies against phase I and II antigens are usually considered as the methods of choice for epidemiological screening of the ruminants [12, 13].

In India, coxiellosis or Q fever remains a neglected zoonosis and lack appropriate clinical attention mainly due to the lack of epidemiological data and diagnostics, poor disease surveillance, and lack of disease awareness even among the public health professionals including veterinarians and clinicians [10, 11, 14, 15]. The objectives of the present study were to investigate *C*. *burnetii* infection in bovines (cattle and buffaloes) from different geographical regions of India and to identify the potential risk factors at farm level.

## Materials and methods

### Study design

The present study was carried out at four different geographical regions of India, Uttar Pradesh, Rajasthan, Chhattisgarh and Haryana. A total of 711 bovines [cattle (n = 543) and buffaloes (n = 168)] from eight farms were screened for coxiellosis. The required consents were obtained from the farm owners to participate in the study and the study did not involve any endangered or protected species. All the adult female animals of the age group ≥ 2 years were included in the study. The location details of the selected farms and number of bovines screened are presented in Table 1. A comprehensive review of literature was conducted to develop a questionnaire comprising of 21 parameters pertaining to animal health and farm management practices (S1 Table) for identification of potential risk factors for coxiellosis in ruminants [1, 5, 7, 11]. Epidemiological data was collected through the questionnaire while collecting the samples (S1 Table).

**Table 1:** Sampling details for investigating of *C*. *burnetii* infection in bovines from different geographical regions of India.

Study Area	Targeted Farms	Animals Screened
Uttar Pradesh	Farm 1	182 Cattle
	Farm 2	74 Cattle
Rajasthan	Farm 3	208 Cattle
Chhattisgarh	Farm 4	34 Cattle
	Farm 5	45 Cattle
	Farm 6	24 Buffaloes
Haryana	Farm 7	114 Buffaloes
	Farm 8	30 Buffaloes
Total	08	Cattle: 543
		Buffaloes: 168

### Sampling procedure

The blood sample (10 ml) from each animal was collected aseptically as a part of routine farm check-up for brucellosis control program under the supervision of veterinarians following the national ethical guidelines. The aliquots from these samples were collected aseptically into 5 ml capacity tubes of BD Vacutainer^®^ spray-coated with K2EDTA tube for blood analysis and BD Vacutainer^®^ SST II Advance (Becton Dickinson, USA) for serum separation. The collected samples were transported in coolant boxes from the place of their collection to the laboratory. The whole blood samples were stored at -20°C until further analysis. In order to collect sera, the clot activator tubes containing blood samples were kept at 4°C and then centrifuged within 12 h of collection at 2500 x *g* for 10 min for serum separation. The serum samples were stored at −20°C until further use for serological studies.

### Detection of *C*. *burnetii* by PCR assays

The DNA was extracted from blood samples by using DNeasy Blood and Tissue kit (Qiagen, USA), as per the manufacturer’s instructions. The concentration and purity of DNA was determined by measuring the optical density at both 260 nm and 280 nm using BioSpectrometer (Eppendorf, Germany). The purified DNA was stored at –20°C for subsequent analysis.

The extracted DNA samples were tested for *C*. *burnetii* employing the trans and the *com1* genes. The details of the primers used for PCR assays are given in S2 Table.

In brief, trans-PCR assay targeting the transposable repetitive insertion sequence *IS1111* of *C*. *burnetii* was performed as per the protocol described earlier [10, 14]. Similarly, PCR targeting the *com1* gene was performed as described earlier [16]. The DNA of *C*. *burnetii* Nine Mile phase 1 (strain RSA 493) was used as a positive control in both the PCR assays.

### ELISA

The bovine serum samples were screened by using indirect-ELISA kit (Bio-X Diagnostics, Rochefort, Belgium) for the detection of one or both anti-*C*. *burnetii* phase I and phase II IgG antibodies as per the instructions provided by manufacturer. The sensitivity and specificity for the ELISA kit used in the study were reported to be 100% and 99.49%. All samples were tested in duplicate and the optical density (OD) of the samples were averaged and corrected by subtracting the OD of the negative control. The results were interpreted by calculating the coefficient of serum samples as per the given formula:

$$Sample’s\;Coefficient=\frac{\left(OD\;of\;sample-OD\;of\;negative\;serum\right)}{\left(OD\;of\;positive\;serum-OD\;of\;negative\;serum\right)}\;x\;100$$

The results of assays were dichotomized as positive or negative based on the cut-offs summarized by the manufacturer. A sample was considered negative, if its coefficient was less than 37%, and positive, if the coefficient was ≥ 37%.

### Statistical analysis

The univariable analysis of risk factors were carried out by calculating odds ratio by using Epi Info™ 7 (CDC) software for the collected farms data (S1 Table). The factors taken into consideration were farm-level characteristics (mean age of animal in a farm, average milk production per lactation in a farm, grazing system, quarantine, floor spacing, calving practices followed at farm, disposal of placenta, disinfection practices and hygiene level of farm workers) and animal-level characteristics [bovine species, breed of cattle, individual animal age, milk production per lactation, mastitis and reproductive disorders].

The data obtained from the questionnaire and diagnostic results were analyzed using SPSS version 24.0 (SPSS Inc., IBM, NY, USA). Exempting the risk factors which represent common management conditions at farm level such as grazing system, quarantine for animals, floor spacing per animal, all other factors with p value of less than 0.2 in univariable analysis and collinearity (r) of less than 0.6 were considered for multivariable logistic regression analysis. Spearman’s rank correlation test was used to assess the collinearity between covariate risk factors. The prevalence of *C*. *burnetii* was considered as binary response variable. The results were presented as odds ratio and 95% confidence interval with p value of less than 0.05 considered as statistically significant.

## Results

### Apparent prevalence of *C*. *burnetii*

The results of PCR assays (trans-PCR and com1-PCR) and ELISA for the targeted farms are presented in Table 2. An overall apparent prevalence (positive for PCR and/or ELISA) of coxiellosis among cattle and buffaloes were 24.5% (133/543) and 8.9% (15/168), respectively.

**Table 2:** Results of PCR assays and ELISA screening for coxiellosis in bovines.

Study area	Targeted farms	Animals	Blood samples		% ELISA positivity
			% trans-PCR positivity	% com1-PCR positivity	
Uttar Pradesh	Farm 1	182 Cattle	12.6% (23/182)	9.3% (17/182)	23.1% (42/182)
	Farm 2	74 Cattle	6.8% (5/74)	6.8% (5/74)	16.2% (12/74)
Rajasthan	Farm 3	208 Cattle	6.7% (14/208)	4.3% (9/208)	14.9% (31/208)
Chhattisgarh	Farm 4	34 Cattle	5.9% (2/34)	5.9% (2/34)	14.7% (5/34)
	Farm 5	45 Cattle	4.4% (2/45)	4.4% (2/45)	13.33% (6/45)
	Farm 6	24 Buffaloes	4.2% (1/24)	4.2% (1/24)	12.5% (3/24)
Haryana	Farm 7	114 Buffaloes	0 (0/114)	0 (0/114)	9.6% (11/114)
	Farm 8	30 Buffaloes	0 (0/30)	0 (0/30)	0 (0/30)
Total		Cattle: 543	8.5% (46/543)	6.5% (35/543)	17.7% (96/543)
		Buffaloes: 168	0.06% (1/168)	0.06% (1/168)	8.3% (14/168)

In cattle, the detection of *C*. *burnetii* DNA in blood samples by the trans-PCR [8.5% (46/543)] was significantly higher than com1-PCR assay [6.5% (35/543)] (p<0.05). In buffaloes, only one (1/168) blood sample was found to be positive by both the PCR assays.

On screening serum samples by ELISA, the seropositivity observed was significantly higher in cattle (17.7%, 96/543) as compared to buffaloes (8.3%, 14/168) (p<0.05). None of the bovines screened were vaccinated against coxiellosis.

### Analysis of risk factors for coxiellosis

The details of the 21 parameters pertaining to animal health and farm management are provided in S1 Table. On univariable analysis of odds ratio among cattle and buffaloes, the odds for coxiellosis among cattle was 3.31 times higher as compared to buffaloes (Table 3). Further, in order to avoid the confounding effect of species, the data of buffalo farms (Farm 6, 7 and 8) were excluded while analysing the odds ratio for other farm related parameters (mean age of animals, feeding system, floor spacing and floor design).

**Table 3:** Univariable analysis of observed risk factors for coxiellosis.

Parameters	Risk Factors	Test for *C*. *burnetii* (PCR assays and ELISA)		Odds Ratio	95% CI	p-value
		+ ve	- ve			
Species	Buffaloes	15	153	1.00		0.00002
	Cattle	133	410	3.31	1.88–5.82	
Breed of cattle	Jersey	8	37	1.00		
	Sahiwal	7	27	1.20	0.39–3.71	0.99
	Indigenous and cross-bred	43	165	1.20	0.59–2.30	0.81
	Holstein Friesian	16	58	1.28	0.50–3.28	0.79
	Frieswal	59	123	2.22	0.97–5.06	0.08
Mean age of cattle (in ascending order)	2.4 years (Farm 5)	8	37	1.00		Chi-square for non-linearity: p-value = 0.027
	2.8 years (Farm 2)	16	58	1.28	0.50–3.28	
	3.2 years (Farm 4)	7	27	1.20	0.39–3.71	
	3.5 years (Farm 1)	59	123	2.22	0.97–5.06	
	4.8 years (Farm 3)	43	165	1.20	0.59–2.30	
Grazing system	Semi-intensive (Farm 3 and 4)	50	192	1.00		0.08
	Stall feeding (Farm 1, 2 and 5)	83	218	1.46	0.98–2.18	
Quarantine for animals	No (Farm 3, 4 and 5)	58	229	1.00		0.02
	Yes (Farm 1 and 2)	75	181	1.64	1.10–2.43	
Floor spacing/animal	Adequate (Farm 3, 4 and 5)	58	229	1.00		0.02
	Inadequate (Farm 1 and 2)	75	181	1.64	1.10–2.43	
Mastitis (animal level)	Not having mastitis	117	506	1.00		0.0006
	Having mastitis	31	57	2.35	1.45–3.81	
Reproductive disorder (animal level)	No reproductive disorder	102	478	1.00		0.00001
	Presence of reproductive disorder	46	85	2.54	1.67–3.85	

The odds of coxiellosis among different breeds of cattle was non-significant (p-value: > 0.05) (Table 3). Also, the association of coxiellosis and the mean age of the animals at the farm level revealed non-linear relationship (p-value for non-linearity = 0.027). Further, the stall-feeding system among cattle although revealed 1.46 higher odds of coxiellosis as compared to semi-extensive grazing system, however, the association was non-significant (p-value = 0.08) (Table 3).

Contrary to the above observations, the inadequate floor spacing per animal at Farms 3, 4 and 5 exhibited 1.64 higher odds than the other farms (Farm 1 and 2). Also, the history of clinical entities such as mastitis and reproductive disorders at individual animal level among bovines screened were having the odds of 2.35 and 2.54 (p-value: <0.05) for coxiellosis, as compared to apparently healthy animals (Table 3).

The ventilation was found to be sufficient in all the targeted farms, however, other biosecurity conditions of the farms (except for farms 7 and 8) were inadequate. For example, absence of the foot bath disinfection, the presence of rodents, wild birds and the pets contact was evident at the farms. Moreover, the management of manure and disposal of placenta was not as per the standard requirements (except at farms 7 and 8). None of the farms were following proper quarantine procedures and regular floor and farm equipment disinfection procedures. The farm workers were practicing hand-washing either at the end of the day or after the completion of farm operations. None of the farm workers were found to have awareness on coxiellosis. However, the artificial insemination practices, use of calving box and isolation of aborted animals were followed in all the farms except at farm 3.

For multivariable binary logistic regression analysis, the significant variables (at the level of p value < 0.2) in the univariable analyses (Table 3) were included. In logistic regression analysis, only the species and age at individual animal level (years) were found to be associated with *C*. *burnetii* infection in bovines as described in Table 4. The cattle were having 2.27 (95% CL: 1.25–4.12) higher odds for coxiellosis as compared to buffaloes. The increase in age of animals were found to have 1.67 (1.46–1.92) higher odds of the *C*. *burnetii* infection.

**Table 4:** Multivariable binary logistic regression analysis of animal-level risk factors associated with prevalence of *C*. *burnetii* in dairy cattle.

Variables		Odds Ratio (95% CI)	p-value
Species	Cattle	2.27 (1.25–4.12)	0.01
	Buffaloes	1	
Mastitis	Positive	1.38(0.81–2.36)	0.24
	Negative	1	
Reproductive disorders	Positive	1.49 (0.93–2.37)	0.10
	Negative		
Age (animal-level) (years)		1.67 (1.46–1.92)	<0.001

## Discussion

*Coxiella burnetii*, the etiological agent of Q fever or Coxiellosis, is a zoonosis affecting variety of animals including ruminants. Information on the epidemiology of *C*. *burnetii* in animals is inadequate in India. The study appears to be first of its kind in India which involves bovines from different states to assess the apparent prevalence of coxiellosis at farms including the associated risk factors. Evidence of *C*. *burnetti* infection was sought in bovines (cattle and buffaloes) by the detection of bacterial DNA and the serology.

In this study, an overall apparent prevalence (positive for PCR and/or ELISA) of coxiellosis observed was 24.5% in cattle and 8.9% in buffaloes. A review comprising qualitative assessment of 69 publications indicated the detection of *C*. *burnetii* infection in all 5 continents (Africa, America, Asia, Europe and Oceania) with wide range of host species and median prevalence of *C*. *burnetii* infection among cattle was reported as 20% at the animal level [2]. In a recent cross-sectional serosurvey from Italy on C. *burnetii* in apparently healthy cattle (n = 2210), the prevalence at the animal-level was observed as 12.0% [17]. Globally, the epidemiology of coxiellosis in water buffaloes (*Bubalus bubalis*) is largely unknown [18]. In Egypt, 19.3% of cattle were found to be seropositive for coxiellosis as compared to 11.2% of buffaloes [19]. In a study from Punjab State of India, the overall prevalence was observed to be higher in cattle (8.7%) as compared to buffaloes (4.3%) [15]. However, in Iran, milk samples revealed higher prevalence of anti-*C*. *burnetii* antibodies among buffaloes (19.3%) as compared to cattle (14.6%) [20]. Previous studies from India reported prevalence of coxiellosis in bovines ranging from 05.55% to 29.9% [10, 11, 14, 15].

The *C*. *burnetii* detection rate by trans-PCR (8.5%) was significantly higher (p<0.05) as compared to com1-PCR (6.5%) among samples collected from cattle. The difference in the sensitivity of these PCR assays can be attributed to the reported higher sensitivity of *IS1111*, a multi-copy gene having 7–110 copies per isolate of *C*. *burnetii* [21], as compared to single-copy *com1* gene [22]. Similar observations with regard to the sensitivity of trans-PCR and com1-PCR have been reported in recent studies, wherein these tests could detect the pathogens in 63% and 30% [22], 17.14% and 10% [23] of clinical samples, respectively. In a systematic review on epidemiology of *C*. *burnetii* in Iran, a higher prevalence was observed in PCR assays based on the *IS1111* gene (11%) as compared to the *com1* gene (4%) [24].

The ELISA revealed higher positivity for anti-*C*. *burnetii* antibodies in cattle (17.7%) as compared to the trans-PCR (8.5%) and com1-PCR (6.5%) assays (Table 2). A total of 101 bovines were found to be positive in ELISA but negative for PCR assays, which might be attributed to the enduring immunological response of these animals to *C*. *burnetii* that has been correlated with the elimination of the pathogen [25]. The PCR positivity of 38 bovines that showed negative result in ELISA can be attributed to the early acute phase of the infection [9]. The positivity among nine cattle in both PCR as well as ELISA in the present study emphasise an active circulation of the pathogen within the herd, which has also been reported earlier [9, 26].

A total of 21 parameters pertaining to animal health and farm management practices were assessed (S1 Table). On univariable analysis, the cattle were found to have 3.31 higher odds of coxiellosis as compared to buffaloes (Table 3). In addition, the multivariable binary logistic regression analysis also depicted that cattle were having 2.27 higher odds for coxiellosis as compared to buffaloes (Table 4). Earlier, it has been opined that buffaloes might be less susceptible to *C*. *burnetii* infection [27]. However, further studies are needed to estimate the resistance of buffaloes for *C*. *burnetii* infection. The low seropositivity for coxiellosis coupled with non-detection of pathogen at Farms 7 and 8 suggest a low level of *C*. *burnetii* infection on both farms which emphasized the importance of biosecurity measures and good animal husbandry practices which were routinely practised at both the farms (S1 Table).

The susceptibility of different breeds of cattle was found to have non-significant association for coxiellosis (p>0.05). It has been reported that the risk of a cow being seropositive for coxiellosis could vary among the breeds, for example, a higher risk in Danish Holstein than Jersey cows [28, 29]. In present study, the Holstein Friesian and Frieswal have 1.28 (95% CL: 0.50–3.28) and 2.22 (95% CL: 0.97–5.06), respectively, higher odds for coxiellosis as compared to Jersey breed but had non-significant association. However, the genotypic variations among the breeds with regard to coxiellosis need to be investigated in depth before arriving at any conclusion.

The association between mean age of animals at the farm and coxiellosis were having non-linear relationship. However, the old animals were found to have higher odds for coxiellosis in univariate as well as multivariable binary logistic regression analysis (Tables 3 and 4). Earlier, the older animals have been reported to have increased odds of getting infected, most often after the first calving [28, 30] and increasing of age might be associated with higher probability of being exposed to the pathogen [17].

The stall-feeding system among cattle was found to have 1.46 higher odds of coxiellosis as compared to semi-extensive grazing system, however, both feeding system were not associated significantly (p-value: 0.08). In earlier studies, high seroprevalence of coxiellosis in farm with intensive management system have been reported [29, 31]. It has been reported that after the pathogen entry coupled with poor biosecurity practices, the animals in intensive system are at greater risk as compared to the extensive system due to more time of exposure that could lead to direct or indirect transmission in the barn environment [29]. However, the free grazing herds on wider pastures have also been reported to have a higher risk for coxiellosis, due to the possibility of high contact rates with infected animals from different herds [17].

The inadequate floor spacing (Farm 1 and 2) is found to have 1.64 higher odds for coxiellosis as compared to farms having adequate floor spacing. It has been reported that an increasing animal density could result in a higher probability of being directly exposed to *C*. *burnetii* during the parturition or infectious abortions [17].

The mastitis and reproductive disorders among bovines were found to have higher odds for coxiellosis in both univariable (statistically significant) and multivariable binary logistic regression analysis (statistically non-significant) as compared to apparently healthy animals (Tables 3 and 4). Coxiellosis in cattle usually remains asymptomatic [8], however, it has been reported to be associated with subclinical mastitis [32], sporadic reproductive problems such as abortion [8, 33] and metritis [34].

The biosecurity conditions of all the farms (except farm 7 and 8) were found to be not adequate. The importance of quarantine and other biosecurity measures for coxiellosis has been discussed in recent studies [11, 35]. However, in this study, none of the dairy farms were following standard quarantine measures for animals, except at farms 7 and 8, wherein the imported animals were observed for fever, diarrhoea, mastitis and brucellosis for 1–2 days. In India, there is lack of knowledge on coxiellosis among farming community and the disease is also not included in the list of differential diagnosis of abortion cases. Besides this, the foot bath disinfections were not placed at the entrance of the farms, and the presence and contact of rodents, wild birds and pets were evident with farm animals. It has been reported that poor biosecurity practices including the easy access of infected stray or wild animals including birds and wild cats to the farm can be potential sources of infection [3, 36]. Moreover, the management of livestock manure and disposal of placenta were not as per the standard requirements (except farms 7 and 8). The aborted material of infected ruminant has been opined to serve as an important source of infection [37]. The zoonotic potential of the manure has been reported with higher incidences of the Q fever in humans around contaminated farms [38]. It has also been advised that the hygienic measures focusing on the calving practices (e.g., destruction of placentae, specific calving box cleaned and disinfected after each calving period) and the management of manure (e.g., treatment of manure and limiting its wind-borne spread) can be implemented in infected herds to reduce the disease burden [34]. Additionally, the artificial insemination practice was followed in all the farms under investigation, however, there was no evidence of screening the semen for *C*. *burnetii*. The *C*. *burnetii* has been considered among the list of pathogens having the potential to transmitting the infection through the contaminated semen in bovines [39]. None of the farms were following regular floor and farm equipment(s) disinfection procedures. The hand-washing frequencies of the farm workers were inadequate and none were found to have awareness on coxiellosis. The hygiene precautions taken by veterinarians and farm workers, i.e. changing boots and/or clothes have been reported to significantly reduce the risk of coxiellosis [29, 40]. In addition, in earlier studies hygiene related factors were indulged with higher seroprevalence of *C*. *burnetii* infection among high-risk population [41].

The present study has some limitations. In the view of logistic constraints and difficulty in motivating the farm owners to participate in the study for screening their animals for a disease which is unknown for them, we could target eight farms from four different states of India in order to address the geographical distribution of the disease. The targeted farms were having apparently healthy animals and without any history of previous screening and vaccination for coxiellosis, however, the targeted sampling could have biased our calculation of prevalence at animal and farm level due to contagious nature of the disease. Furthermore, the recent description of *Coxiella*-like bacteria (CLB), which are closely related but genetically distinct from *C*. *burnetii*, have posed a diagnostic dilemma in terms of cross-reactivity for many of the *C*. *burnetii* specific genes, including the *IS1111*. However, in the present study, we have targeted only bovines, where the trans-PCR is considered as one of the most sensitive method for epidemiological screening. However, further large scale multi-centric studies need to be undertaken in order to elucidate the epidemiological impact of coxiellosis in animals and public health in Indian settings.

In conclusion, in the present study, a significantly higher prevalence of coxiellosis was observed in cattle (24.5%) as compared to buffaloes (8.9%). The trans-PCR was found to be more sensitive assay as compared to the *com-1* PCR to detect *C*. *burnetii* from blood samples of bovines. The poor correlation between PCR and ELISA suggested that a combination of PCR along with serological test(s) is required to assess the true status of coxiellosis. While analyzing the risk factors, the prevalence of *C*. *burnetii* was found to be influenced mainly by breed, reproductive disorders, mastitis and other herd management practices. The absence of vaccination for coxiellosis among bovines in India, the lack of biosecurity and farm hygiene procedures could be the important factors in the introduction and subsequent transmission of the pathogen in between animals and to the farm workers. Further, it is important to implement a robust surveillance system based on a ‘One Health’ approach. There is need to include coxiellosis in the list of differential diagnoses while investigating reproductive problems in dairy cattle and mass awareness is must among farmers and other at-risk occupational groups.

## Supporting information

S1 Table Parameters pertaining to animal health and farm management on targeted farms. (DOCX)

S2 Table Details of the primers used for the detection of *C*. *burnetii* from blood samples of bovines. (DOCX)
