# Supplementary material for: Apparent prevalence and risk factors of coxiellosis (Q fever) among dairy herds in India
Source: PLoS One. 2020 Sep 15;15(9):e0239260. doi: 10.1371/journal.pone.0239260 (PMC7491716; doi:10.1371/journal.pone.0239260)
Supplement: S1 Table — (DOCX) [file pone.0239260.s001.docx]

**S1 Table: Parameters pertaining to animal health and farm management on targeted farms**

| Parameters | Farm 1 | Farm 2 | Farm 3 | Farm 4 | Farm 5 | Farm 6 | Farm 7 | Farm 8 |
| --- | --- | --- | --- | --- | --- | --- | --- | --- |
| A. Herds characteristics: |  |  |  |  |  |  |  |  |
| 1. Species and Breed   (Indigenous/exotic breed/ Hybrid) | Cattle: Hybrid (Frieswal) | Cattle: Exotic (Holstein Friesian) | Cattle: Indigenous and local cross-bred | Cattle: Sahiwal | Cattle: Jersey | Buffaloes:  Jafarabadi, Murrah | Buffaloes:  Murrah | Buffaloes:  Murrah |
| 1. Age (Mean) | 3.5 years | 2.8 years | 4.8 years | 3.2 years | 2.4 years | 4.2 years | 3.7 years | 4.5 years |
| 1. Grazing system practiced   (Extensive/Semi-extensive/Stall feeding) | Stall feeding | Stall feeding | Semi-intensive | Semi-intensive | Stall feeding | Stall feeding | Stall feeding | Stall feeding |
| B. Housing Characteristics: |  |  |  |  |  |  |  |  |
| 1. Quarantine for purchased animals | Absent | Absent | Absent | Absent | Absent | Absent | Not properly followed | Not properly followed |
| 1. Floor spacing/animal (Adequate: cattle ≥ 3.5 m^2^; buffalo ≥ 4 m^2^) | Not adequate | Not adequate | Adequate | Adequate | Adequate | Adequate | Adequate | Adequate |
| 1. Farm biosecurity | Not adequate | Not adequate | Not adequate | Not adequate | Not adequate | Not adequate | Good biosecurity | Good biosecurity |
| 1. Ventilation (Good/Poor) | Good | Good | Good | Good | Good | Good | Good | Good |
| 1. Management of livestock waste/manure | Within farm premises | Within farm premises | Within farm premises | Within farm premises | Within farm premises | Within farm premises | Away from farm | Away from farm |
| C. Milk related traits: |  |  |  |  |  |  |  |  |
| 1. Average milk production (litres/lactation) | 2710 | 3200 | 915 | 2450 | 2848 | 2142 | 2468 | 3150 |
| 1. Animal-level mastitis (as per farm records) | 12.09% (22/182) | 8.11% (6/74) | 25.96% (54/208) | 5.88% (2/34) | 4.44% (2/45) | Nil | 1.75% (2/114) | Nil |
| D. History of animal-level reproductive disorders  (as per farm records) | 26.37% (48/182) | 4.05% (3/74) | 34.61% (72/208) | 2.94% (1/34) | 4.44% (2/45) | 4.17% (1/24) | 2.63% (3/114) | 3.33% (1/30) |
| E. Type of reproductive service  (Artificial insémination/ Natural service) | Artificial insemination | Artificial insemination | Mix (Artificial insemination and Natural service) | Artificial insemination | Artificial insemination | Artificial insemination | Artificial insemination | Artificial insemination |
| F. Calving practices: |  |  |  |  |  |  |  |  |
| 1. Use of calving box or in open | Calving box | Calving box | In open | Calving box | Calving box | Calving box | Calving box | Calving box |
| 1. Isolation of aborted animal | Yes | Yes | No | Yes | Yes | Yes | Yes | Yes |
| G. Disposal of placenta: |  |  |  |  |  |  |  |  |
| 1. Place of disposal | Within farm premises | Within farm premises | Within farm premises | Within farm premises | Within farm premises | Within farm premises | Away from farm premises | Away from farm premises |
| 1. Method of disposal (use of disinfectant?) | Discard on ground without disinfection | Discard on ground without disinfection | Discard on ground without disinfection | Discard on ground without disinfection | Discard on ground without disinfection | Buried under the ground without disinfection | Buried under the ground without disinfection | Buried under the ground without disinfection |
| H. Vaccination status | None | None | None | None | None | None | None | None |
| I. Disinfection practices followed: |  |  |  |  |  |  |  |  |
| 1. Floor disinfection | Once in a month | Weekly | Rare | Rare | Rare | Weekly | Weekly | In 2-3 days |
| 1. Hands disinfection | At the end of the day | At the end of the day | At the end of the day | At the end of the day | At the end of the day | After farm operations | After farm operations | After farm operations |
| 1. Farm equipment’s disinfection | Occasional | Occasional | Rare | Rare | Rare | Rare | Weekly | In 2-3 days |
| J. Farm workers knowledge on coxiellosis | None | None | None | None | None | None | None | None |
