# Supplementary material for: Apparent prevalence and risk factors of coxiellosis (Q fever) among dairy herds in India
Source: PLoS One. 2020 Sep 15;15(9):e0239260. doi: 10.1371/journal.pone.0239260 (PMC7491716; doi:10.1371/journal.pone.0239260)
Supplement: S2 Table — (DOCX) [file pone.0239260.s002.docx]

**S2 Table**: Details of the primers used for the detection of *C. burnetii* from blood samples of bovines

| **Target gene** | **Primer** | **Primer sequence 5’ – 3’** | **Product size (bp)** | **Reference (s)** |
| --- | --- | --- | --- | --- |
|  |  |  |  |  |
| *IS1111* | trans 1 | TAT GTA TCC ACC GTA GCC AGT C | 687 | Lorenz *et al*., 1998  Berri *et al.,* 2000 |
|  | trans 2 | CCC AAC AAC ACC TCC TTA TTC |  |  |
| *com1* | com1 | AGT AGA AGC ATC CCA AGC ATT G | 501 | Zhang *et al.,* 1998 |
|  | com2 | TGC CTG CTA GCT GTA ACG ATT G |  |  |
